# Description of bone health in adolescents and young persons with Klinefelter syndrome – results from a pilot study

**DOI:** 10.1186/s40348-024-00182-w

**Published:** 2024-09-17

**Authors:** Julia Spiekermann, Jakob Höppner, Eliena Ibnukhsein, Kathrin Sinningen, Beatrice Hanusch, Cordula Kiewert, Heide Siggelkow, Corinna Grasemann

**Affiliations:** 1 Department of Pediatrics, Division of Rare Diseases, University Hospital of Pediatrics and Adolescent Medicine, Katholisches Klinikum Bochum, Member of ENDO ERN, Ruhr-University Bochum, Alexandrinenstraße 5, 44791 Bochum, Germany; 2 Center for Rare Diseases Ruhr (CeSER), Ruhr-University Bochum and Witten/Herdecke University, Bochum, Witten, Germany; 3 University Hospital of Pediatrics and Adolescent Medicine, St. Josef-Hospital, Ruhr-University Bochum, Bochum, Germany; 4 Division of Pediatric Endocrinology, Department of Pediatrics II, Member of ENDO ERN, University Hospital Essen, University of Duisburg-Essen, Essen, Germany; 5 Clinic of Gastroenterology, Gastrointestinal Oncology and Endocrinology, University Medical Center Göttingen, Göttingen, Germany and MVZ Endokrinologikum Göttingen, Göttingen, Germany

**Keywords:** Klinefelter syndrome, XXY, Bone health, Bone health index, Adolescence, Children

## Abstract

**Background:**

In adults with Klinefelter syndrome (KS), impaired bone health with reduced bone mineral density (BMD) has been described even in the presence of testosterone replacement therapy. The aim of the present study was to characterize bone health in young patients with KS.

**Patients and methods:**

20 participants aged 16.10 ± 4.28 years with KS (7 with testosterone replacement therapy) were included in the KliBONE study (DRKS 00024870). Medical history, clinical, radiographic and biochemical parameters of bone health and metabolism were obtained. Radiographic bone health index (BHI) was assessed via automated digital X-ray radiogrammetry of the left hand or via dual energy X-ray absorptiometry (DXA) of the lumbar spine and left femur in participants *≥* 16 years. Peripheral blood mononuclear cells were differentiated into osteoclasts and quantified in 7 participants and 7 healthy controls.

**Results:**

Mean BHI SDS was − 1.42 ± 1.22 and mean BMD z-score at the lumbar vertebrae (L1-4) was − 0.92 ± 1.00. 25-OH-vitamin D levels < 20 ng/ml were detected in 8/20. Other parameters of bone metabolism (bone-specific alkaline phosphatase, PTH, ß-crosslaps and osteocalcin) were within age-appropriate reference ranges. Serum leptin SDS was elevated (mean 2.15 ± 1.19). The number of osteoclasts in participants with KS did not differ from that of controls.

**Conclusion:**

BHI SDS and BMD z-scores were lower than expected in young individuals with KS despite age-appropriate bone turnover markers and no apparent pathology in osteoclast differentiation. The cause of the early-onset bone phenotype requires further investigation.

**Supplementary Information:**

The online version contains supplementary material available at 10.1186/s40348-024-00182-w.

## Introduction

Klinefelter syndrome (KS) is caused by an extra X chromosome in male individuals, resulting in the karyotype 47,XXY (and variants) and affects approximately one in 500 men [1, 2]. In all non-mosaic forms gonadal failure develops, usually detectable around mid to late puberty and results over time in hypergonadotropic hypogonadism with small, firm testes, azoospermia and infertility [1]. Additionally, men with KS have orginally been described as individuals with a tall and feminine body habitus and marked gynecomastia [1]. However, the clinical spectrum is much wider and the presentation is highly heterogeneous which leads to a diagnostic delay of KS especially in childhood. It is estimated that only up to 40% of the affected men will receive the diagnosis of KS in their lifetime and only about 20% are being diagnosed during childhood and adolescence [2, 3]. A wide variety of organ systems has been reported to be affected in KS, resulting in increased morbidity and a reduced quality of life due to varying levels of impairment in cardiovascular, endocrine, metabolic, cognitive, and neuropsychological health [1, 4, 5]. 

Ferlin et al. reviewed that adult men with KS have an increased prevalence of metabolic bone disease and reduced bone mineral density (BMD). The combined rate of osteopenia and osteoporosis in men with KS is increased up to 40% compared to men without extra X chromosomes of the same age [6]. The underlying mechanism of the reduced bone mineral density is yet unclear and cannot be attributed to hypogonadism only, as osteoporosis and osteopenia also occur in men with KS with normal or normal-low testosterone levels [7, 8]. Data regarding the influence of testosterone replacement therapy (TRT) on bone mass, are discussed controversially. Late initiation of TRT does not appear to normalize BMD in adults with KS, but early initiation (before the age of 20 years) seems to normalize BMD [9, 10]. 

Pediatric studies on bone health in KS are rare but suggest that cortical bone mass or BMD are already impaired at a prepubertal age, and thus before the onset of hypogonadism [11]. An unfavorable body composition with increased adipose tissue, an increased prevalence of type 2 diabetes and metabolic syndrome as well as reduced motivation for exercise, have been described in children and young adults and pathogenetically discussed [12–14]. Aksglaede et al. described significantly increased body fat mass despite normal lean body mass and BMI for age, suggesting that an unfavorable muscle-fat ratio may already be present in childhood [15]. Physical activity has a significant influence on bone health. In a recent study by our group, we identified a marked impairment of physical fitness in a small group of adolescents with KS [4], echoing the findings of Skakkebæk et al. who showed a tendency towards physical inactivity in KS adults, [14] whereas others found no difference in the quantitative amount of exercise between patients with KS and controls [16]. 

Reduced levels of vitamin D in KS seem to be frequent in children as well as in adults [16, 17]. Recent data also identified a reduced BMD in boys with KS compared to boys with 46,XY [18]. However, changes in bone health have not been investigated in bone biopsies or on a cellular level, so far. The aim of this study was to characterize bone health in children and adolescents with KS in depth in a pilot cohort.

## Materials and methods

### Cohort

Individuals with KS were enrolled from April 2021 to March 2023 in the KliBONE study (DRKS No.: DRKS00024870). Participants were recruited during annual screening visits and in cooperation with national patient groups for 47,XXY [19, 20]. Inclusion criteria were a genetically confirmed diagnosis of KS, age between 9 and 25 years and signed informed consent from participants and/or their legal guardians if applicable. The study protocol was approved by the Ethics committee of the Medical Faculty, Ruhr-University Bochum (#21-7164) and the study was conducted in accordance with the principles of the Declaration of Helsinki.

### Clinical parameters

Clinical and anamnestic parameters were assessed as previously described [21, 22]. In brief, the following parameters were obtained: age at study participation, age at diagnosis, birth weight and length, parental heights, current medication, medical history including detailed information on the start, frequency, and dose of testosterone replacement therapy if applicable. Standing height (in cm) was measured to two decimal places using a wall-mounted stadiometer (Ulmer Stadiometer, Busse Design, Elchingen, Germany), weight (in kg) was measured to two decimal places as well (Seca, Hamburg, Germany). BMI (kg)/(height in m)^2 was calculated and z-scores for BMI, height and weight based on the KiGGS data (9–18 years) and the WHO data (> 18 years) [23, 24] were calculated. The pubertal status (Tanner stages) and the determination of testicular volume using a Prader orchidometer were assessed by experienced pediatric endocrinologists. Body impedance was measured using the TANITA Body composition analyzer (Model DC-360, TANITA Europe B. V., Amsterdam, the Netherlands). No participants with mosaic chromosomal status were included.

### Biochemical tests

As previously described [4], serum, plasma and spot-urine aliquots were assessed in the central laboratory of the St. Josef-Hospital Bochum and at the MVZ Dr Eberhard & Partner Dortmund, Germany. The respective samples were obtained in the mornings (before 10 am, non-fasting) and then stored at -80 °C until the analysis. The following parameters were assessed: hemoglobin (g/dl); thyroid-stimulating hormone; TSH (uIE/ml); free triiodothyronine, fT3 (pg/ml); thyroxine, fT4 (ng/dl); calcium (mmol/l); phosphate (mg/dl); 25-OH vitamin D ( 25OHD, ng/ml); 1,25-(OH)_2_ vitamin D (pg/ml); total serum alkaline phosphatase, TSAP (U/l); bone specific alkaline phosphatase, BAP (ug/l); parathyroid hormone, PTH (pg/ml); osteocalcin, OC (ng/ml); insulin-like growth factor-1, IGF-1 (ng/ml); beta-crosslaps, CTX (pg/ml); leptin (ng/ml); free and total testosterone (ng/ml); follicle stimulating hormone, FSH (IU/ml) and luteinizing hormone, LH (IU/ml). In addition, deoxypyridinoline, DPD (mg/g creatinine), calcium and creatinine were measured in spot urine and the calcium: creatinine ratio (in mg/mg) was calculated subsequently. Leptin SDS was calculated using the reference values of Blum et al. [25]. We provide information of inter and intra assay precision in suppl. Table 3.

### Dual X-Ray absorptiometry (DXA) and Bone Health Index (BHI)

Bone mineral density was assessed using DXA in six patients aged 16–25 years (Lunar Prodigy, GE-Healthcare, Madison, WI, USA). The BMD was assessed at the lumbar spine (L1-L4; anteroposterior view) and at the total left femur. Z-scores were calculated for the lumbar spine measurements based on normative values measured by the manufacturer for the corresponding age without correction for height.

The Bone Health Index (BHI) was obtained from a conventional radiograph of the left hand from an anterior-posterior view in 14 individuals aged 9–17 years. Digital images were saved in DICOM (Digital Imaging and Communications in Medicine) format and analyzed for skeletal age, BHI and BHI SDS using BoneXpert software (BoneXpert version 2, Visiana, Holte, Denmark) as previously described by Thodberg et al. [26]. As a radiogrammetric method, the BHI describes bone mass as a function of the cortical thickness of three metacarpals and the width and length of the metacarpals. BHI standard deviations are calculated based on a large Caucasian reference cohort. BHI SDS has been shown to correspond to lumbar BMD SDS using DXA in pediatric cohorts [26–28]. 

### Questionnaires

To obtain information about fractures and skeletal-associated pain, participants completed a specific questionnaire which was adapted from the questionnaire of the Child and Adolescent Health Survey (KiGGS) from the Robert Koch Institute (RKI) [29]. The questions of the RKI are based on the pain-related questionnaire according to Perquin et al. [30, 31]. Based on 35 questions, the 3-month pain prevalence, localization, frequency of occurrence, intensity and the first occurrence of pain were recorded. In addition, questions were asked about individual factors related to the development, consequences, and negative impact of pain to determine whether increased pain limits movement and exercise in this cohort. In a second step, information on the number and type of fractures in the previous history was asked [31]. 

### Osteoclast differentiation & TRAP-staining

Peripheral blood mononuclear cells (PBMCs) were isolated from EDTA whole blood samples by density centrifugation from 7 study subjects and 7 matched controls. PBMCs were seeded in triplicates at a density of 1 × 10^6^/cm^2^ in α-MEM (Pan Biotech, Aidenbach, Germany), 10% FBS (Gibco™, ThermoFisher, Darmstadt, Germany), 1% Pen-Strep (Pan Biotech, Aidenbach, Germany), and 25 ng/ml macrophage colony-stimulating factor (M-CSF; PeproTech, Hamburg, Germany). After 3 days, 50 ng/ml receptor activator of NF-κB ligand (RANKL; PeproTech, Hamburg, Germany) was added additionally. Cells were incubated at 37 °C and 5% CO_2_ with changes of medium every 2–3 days. To assess osteoclast differentiation, cells were stained for tartrate-resistant acid phosphatase (TRAP, Acid Phosphatase Kit 387-A; Sigma-Aldrich, St. Louis, MO) after 14 days of differentiation. Four sections of each well were photographed using a Axiocam 305 color, Zeiss camera. Osteoclasts of all sections were quantified using ImageJ software (Fiji; National Institutes of Health, Bethesda, USA). Multinucleated (≥ 3 nuclei), TRAP-positive cells were counted as osteoclasts.

### Statistical analysis

Statistical analysis was performed using Jamovi 2.3 version 1.6 for Mac (The Jamovi project [2021]) [32]. Data are presented as mean ± standard deviation (SD) of mean, data for osteoclast counts are presented as median and 25th and 75th percentile. Data were tested for normal distribution using the Shapiro-Wilk test. Figures were created using GraphPad Prism version 9.5.1 for macOS (GraphPad Software, San Diego, California USA) [33]. 

## Results

### Participants

Twenty individuals with KS (mean age of 16.10 ± 4.28, range 9.26–25.40 years) were included in this pilot cohort study. The descriptive statistics are shown in Table 1, the individual results are shown in suppl. Tables 1 and 2.

**Table 1:** Characteristics of 20 participants with KS

	Mean ± SD	Range
Age at visit (years)	16.10 ± 4.28	9.26–25.40
Age at diagnosis (years)	6.50 ± 6.59	0–17
Height (z-score)	1.40 ± 1.19	-1.04–4.20
Bodyweight (z-score)	0.91 ± 0.97	-0.52–2.69
BMI (z-score)	0.15 ± 0.33	-2.00–2.50
Fat mass (%, Impedance Scale)	22.93 ± 9.09	9.20–38.30
Testosterone replacement therapy (n/cohort)	7/20	
TANNER Stage 1/2/3/4/5	2/2/2/4/10	
Fractures (n/cohort)	4/20	
3 months general pain prevalence (n/cohort)	9/20	
3 months back pain prevalence (n/cohort)	8/20	
Total BMD L1-L4 (g/cm^2^)	1.05 ± 0.08	0.91–1.16
Total BMD L1-L4 z-score	-0.92 ± 1.00	-1.70–0.90
Total BMD femur left (g/cm^2^)	1.03 ± 0.13	0.84–1.19
Total BMD femur left z-score	-0.63 ± 0.98	-1.50–1.20
BHI SDS	-1.42 ± 1.22	-3.04–0.94

Continuous data are shown as mean ± standard deviation (SD), range (min – max).

BMI - body mass index, BMD – bone mineral density, BHI – bone health index

The pubertal status was determined as following: Tanner 1 = 2; Tanner 2 = 2; Tanner 3 = 2; Tanner 4 = 4; Tanner 5 = 10. 7 participants received a testosterone replacement therapy (testosterone undecanoate 250 mg/3–4 weekly (*n* = 3); testosterone undecanoate 1000 mg/3 monthly (*n* = 1), testosterone gel 25–50 mg/transdermal daily (*n* = 3)). Additional medication included: Lisdexamfetamin (*n* = 2), methylphenidate (*n* = 2) and salmeterol/fluticasone (*n* = 1).

The average body fat percentage was 22.93 ± 9.09%, with 5 participants in the overfat range according to age-appropriate norm values (defined as 95th to 98th percentile for < 18 years according to McCarthy et al. or body fat % 20–25 for ≥ 18 years according to Gallagher et al.) and 5 participants in the obese range (defined as > 98th percentile for < 18 years or body fat % > 25 for ≥ 18 years) [34, 35]. 

One or more fractures had occurred in 4 participants, all of those were fractures of the wrists. Regarding skeletal pain, 9 participants reported general pain and 8 back pain within the last three months.

### Biochemical results

**Fig. 1:**
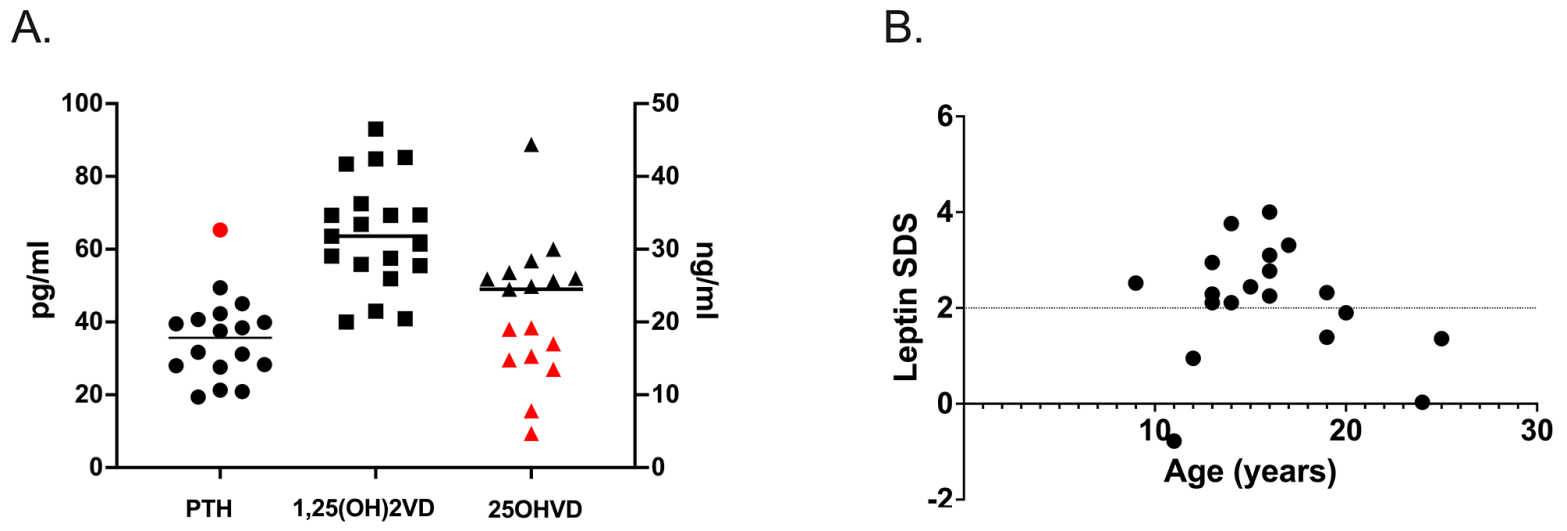
**(A)** Hormonal parameters of calcium-phosphate metabolism; circles: PTH = parathyroid hormone (pg/ml; left y-axis), squares: 1,25(OH)2VD = 1,25 dihydroxy vitamin D (pg/ml; left y-axis), triangles: 25OHD = 25 hydroxy vitamin D (ng/ml; right y-axis); red filling indicate readings outside of the age-appropriate reference range. **(B)** Leptin was elevated (expressed as SDS, *n* = 19); the dotted line represents the upper range of normal

A vitamin D deficiency (serum 25OHD < 20 ng/ml) was present in 8 participants. Three participants supplemented vitamin D3. Serum calcium and phosphate values were unremarkable and serum PTH was within the normal range in all but one participant. Calcium excretion in the urine was in the normal range (Fig. 1A, supp. Table 2).

The osteoanabolic markers osteocalcin (OC) and bone specific alkaline phosphatase (BAP) as well as the bone resorption markers beta-crosslinks and deoxypyridinoline in urine were within age-appropriate norms with one exception (supp. Figure 1, supp. Table 2). Leptin SDS was elevated with a mean of 2.15 ± 1.19 (range − 0.78–4.00; Fig. 1B). Parameters of thyroid function, Cortisol and IGF-1 were within normal ranges. For comprehensive biochemical results refer to supp. Table 2.

### Measurement of BHI/BMD

**Fig. 2:**
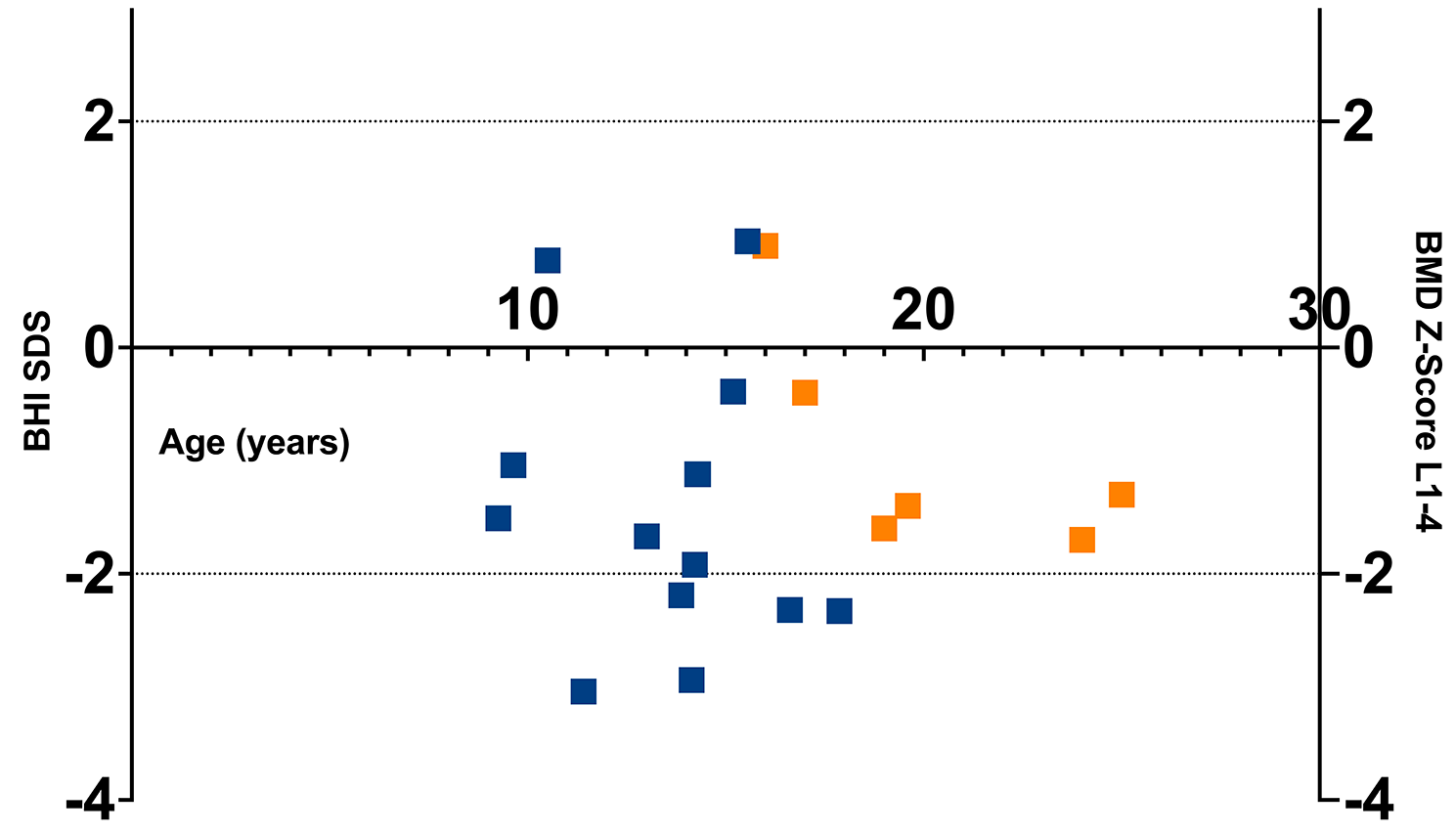
Results for radiographic measurements: Radiogrammatic assessment of the bone health index (BHI) SDS from X-rays of the left hand (*n* = 14; blue squares; blue square at BHI SDS = -1.12 corresponds to the BHI for two participants aged 14 years) and z-scores of bone mineral density (BMD) scans at the lumbar spine (L1-L4) using DXA methodology in 6 participants (orange squares)

The mean BMD z-scores were within the normal range in 6 participants (BMD L1-L4 z-score: -0.92 ± 1.00, range − 1.70–0.90, left femur BMD z-score − 0.63 ± 0.98, range − 1.50–1.20). In participants aged 9 to 17 years (*n* = 14), the BHI SDS was determined and was lower than expected (-1.42 ± 1.22, range − 3.04–0.94, p-value < 0.001) (Table 1; Fig. 2).

### Osteoclast differentiation in vitro

**Fig. 3:**
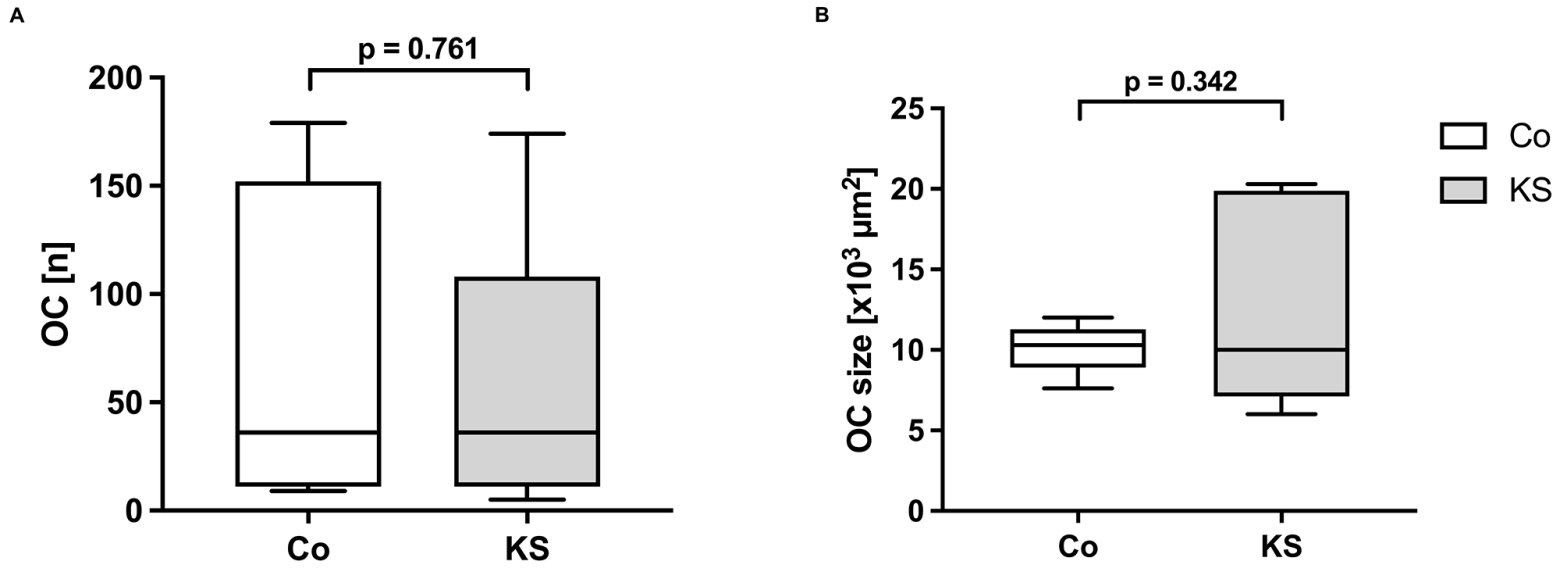
Quantification of osteoclast (OC) number (**A**) and size (**B**) (participants with Klinefelter Syndrome (KS) compared to healthy controls (Co). *n* = 7 per group; analyzed with unpaired t-test (A.) and Welch’s t-test (B.)

The number of osteoclasts did not differ in participants with KS (36 [11, 108]; median [P25, P75]) compared to healthy controls (36 [11, 152]; Fig. 3A). Osteoclasts from participants with KS showed a greater variance in their measured size (10.0 [7.12, 19.9] x10^3^ µm^2^) compared to controls (10.3 [8.92, 11.3] x10^3^ µm^2^). However, these differences were not statistically significant (Fig. 3B).

### Comprehensive assessment

Data on this cohort expand to previously published data on cardiorespiratory fitness during ergometer testing. No statistically significant association between BHI-SDS/BMD Z-score and Fat mass, testosterone levels or maximum workload (Watt) SDS during the ergometer testing was detectable. The relevant items are combined in Table 2.

**Table 2:** Overview of individual results for BHI-SDS/BMD (L1-L4) z-score, pubertal status, markers of adiposity (leptin SDS and fat mass(%)), fitness (hand grip strength and maximum workload on ergometer)

ID	Age	Tanner Stage	BHI SDS/BMD z-score L1-4	Leptin SDS	Fat Mass (%)	Maximum Grip Strength z-score	Maximum Workload z-score	Testosterone Replacement Therapy
5	11.41	2	-3.04	-0.78	29.2	1.8	-2.46	no
18	16.39	4	-2.94	2.77	32.9	-0.21	-2.62	no
19	17.19	5	-2.33	3.31	33.1	0.29	-2.96	no
17	16.61	5	-2.32	3.1	26.6	1.05	-2.03	no
10	13.88	4	-2.19	2.95	19	-0.17	-0.66	no
15	14.22	5	-1.92	2.11	11.7	3.74	0.19	no
6	24.22	5	-1.7	0.03	9.2	0.54	-0.69	yes
16	13.01	4	-1.67	2.11	27.7	1.92	-1.35	no
13	19.93	5	-1.6	1.39	13.9	1.1	-0.58	yes
7	9.26	1	-1.51	2.52	27.8	-1.26	n/a	no
8	19.84	5	-1.4	2.32	15.6	0.77	-0.38	yes
2	25.43	5	-1.3	1.36	35.1	1.83	-0.12	yes
1	14.29	3	-1.12	3.76	29.1	-0.42	-2.49	no
3	13.4	3	-1.12	2.29	15.9	0.57	0.22	no
14	12.45	1	-1.04	0.95	38.3	0.72	-2.09	no
12	20.91	5	-0.4	1.9	25.5	n/a	0.01	yes
9	15.19	4	-0.39	2.44	16.2	-1.15	-1.69	no
20	10.64	2	0.77	n/a	n/a	n/a	n/a	no
11	16.44	5	0.9	4	19.4	1.31	-0.23	yes
4	16.37	5	0.94	2.25	9.4	3.13	-3.04	yes

Data of fat mass, maximum hand grip strength z-score, maximum workload (measured in watts) z-score, testosterone replacement therapy (TRT) and detailed results of the cardiorespiratory tests are published in Spiekermann et al. *‘Cardiorespiratory fitness in adolescents and young adults with Klinefelter syndrome – a pilot study’ (2023)*(4)

Sorted by BHI in ascending order.

## Discussion

Data on bone health and osteopathologies in men with KS have been largely limited to adults. In this cross-sectional study, we examined bone health comprehensively in a small cohort of boys and adolescents. The main finding of our study is a lower-than-expected bone density measured as BMD or BHI, despite age-appropriate bone turnover markers and unremarkable osteoclast differentiation.

While it is well known that skeletal growth and bone mineralization are influenced substantially by genetic background, hormonal factors, an unfavorable body composition and reduced physical activity may also influence bone quality [36, 37]. Hypogonadism in form of low testosterone in men is associated with decreased bone mass [36, 38]. In KS, the onset of gonadal failure is highly variable but usually does not occur before the onset of puberty. Accordingly, previous studies have shown a reduced BMD post puberty and in adulthood [1, 6, 8, 39]. Stagi et al. examined bone health in 40 Italian children and adolescents with KS and found reduced levels of 25-OH vitamin D and bone formation markers such as bone-specific alkaline phosphatase (BAP) and osteocalcin, as well as higher parathyroid hormone (PTH) levels [16]. The influence of testosterone therapy on the skeletal phenotype in boys and adolescents with KS has been discussed controversially, but most recent study results show an improvement in BMD with androgen therapy and provide evidence that testosterone levels may play a role already in prepubertal age [10, 18]. However, the present study suggests that BMD is in the low-normal range already in prepubertal boys and BMD was also reduced in the majority of participants with TRT. These results are in line with a recent report by Krabbe et al. suggesting additional contributors than low testosterone to reduced BMD in KS [18]. In addition, Vogiatzi et al. also showed low cortical bone mass in prepubertal or pubertal boys with KS and further showed that treatment with oxandrolone improved cortical BMD at the hand expressed as BHI SDS after 2 years compared to the placebo group [10]. In summary, these results suggest that the cause of reduced BMD in KS is multifactorial and that testosterone deficiency is relevant, but not the only, factor.

In addition to hypogonadism, vitamin D deficiency and a resulting calcium deficiency is often discussed in poor bone health. Nearly 40% of the individuals studied in this cohort showed a vitamin D deficiency (defined as 25-OH vitamin D levels < 20 nmol/l). However, these numbers are similar to other reports both in childhood and adulthood and may not explain an effect of vitamin D deficiency to the skeletal system [17, 40]. In fact, a secondary elevation of PTH indicating a relevant calcium deficiency, was only observed in 1 participant thus making it unlikely that the low vitamin D levels contribute to the low BMD in a relevant way. Other laboratory markers of bone formation and resorption were mostly within normal ranges in this cohort. Of note, these results contradict those of Stagi et al. who reported reduced BMD as well as reduced levels of bone formation markers (BAP and OC) in prepubertal individuals with KS [16]. 

While the rate and type of the reported fractures were unremarkable in this cohort, about 40% of the participants reported back pain within the last 3 months. Back pain in childhood is considered a potential symptom of vertebral fractures, but participants were asymptomatic during clinical investigation and the lumbar DXA scans were unremarkable. Individuals with KS report musculoskeletal pain more frequently than the general population [5] but it is feasible that the reported back pain is an early manifestation of a bone disease which is more prevalent in KS [6]. 

Physical activity is closely linked to bone health [41–43]. The transmission of mechanical stimuli to the skeletal system mediated by osteocytes induces the maintenance and increase of bone strength [44, 45]. Koedijk et al. reviewed a negative correlation between sedentary behaviour and BMD at the femoral neck in young adults [46]. A Danish study on quality-of-life reported reduced physical activity in adults with KS [14] and is supported by recent findings of our group showing impaired cardiorespiratory fitness as well as increased sedentary behaviour in a small group of adolescents with KS compared to age matched male controls. In that study participants with KS achieved only one tenth of the WHO’s recommended weekly physical activity, a finding that may also have an impact on bone health [4, 47].

Interestingly, leptin levels in this study were elevated on average two standard deviations regardless of age. While the BMI was in the normal range, the percentage body fat was increased on average which may explain the higher leptin levels. As reviewed elsewhere before [48], leptin itself has a controversial effect on bone via two different signaling pathways: Leptin appears to have an osteo-catabolic effect via a central pathway by binding to hypothalamic receptors and activating two sympathomimetic-mediated processes, first by indirectly inhibiting osteoblast proliferation and second by upregulating RANKL expression [49]. In contrast, peripherally leptin interacts with bone marrow stem cells (BMCs), osteoblasts, and osteoclasts, via the leptin receptor [50, 51]. Thereby, it promotes the proliferation of BMCs from the bone marrow and the differentiation into the osteoblastic lineage, and directly inhibits RANKL secretion in osteoblasts [52, 53]. Interestingly, Thomas et al. could show that leptin is positively associated with BMD in women, but not in men [54]. It is feasible that in KS the differentiation of BMCs into the adipose lineage is favoured thereby creating a subnormal osteoblast-lineage.

Considering osteoclasts as a possible factor for increased bone resorption leading to low BMD in KS individuals, the ex vivo assay for differentiation showed no difference between osteoclasts of patients and control subjects regarding number and size of cells. Thus, the question whether an alteration in bone formation could be present and contribute to the lower bone density of people with KS prevails. Subsequent studies should therefore focus on the role of osteoblasts as a potential cause of reduced bone mass in KS.

### Limitations

The study is limited by the small number of participants. Therefore, the generation of larger cohorts is necessary to strengthen the validity. For the clinical parameters published reference values and normative cut offs were used, but no control group established. Longitudinal data are necessary especially in comparison of individuals with/without testosterone substitution. For the development of longitudinal data, we encouraged all of the participants to join the International Registries For Rare Conditions Affecting Sex Development & Maturation (I-DSD, https://sdmregistries.org/i-dsd/). [55].

## Conclusions

Participants with KS had lower than expected bone mineral density readings already at a prepubertal age despite slightly increased height and normal BMI, and normal parameters of bone metabolism. The elevated leptin levels indicate at a potential inverse relationship of bone and adipose tissue in this cohort. However, given the complex and diverse phenotype in individuals with KS, these results underline the theory that the reduced BMD is caused by a multifactorial process which requires further investigations.

## Electronic supplementary material

Below is the link to the electronic supplementary material.

Supplementary Material 1

## Data Availability

No datasets were generated or analysed during the current study.
