# Supplementary material for: Description of bone health in adolescents and young persons with Klinefelter syndrome – results from a pilot study
Source: Mol Cell Pediatr. 2024 Sep 17;11:9. doi: 10.1186/s40348-024-00182-w (PMC11405648; doi:10.1186/s40348-024-00182-w)
Supplement: Supplementary file 1 — Supplementary Material 1 [file 40348_2024_182_MOESM1_ESM.docx]

Supplementary Material

Description of Bone Health in adolescents and young persons with Klinefelter Syndrome – Results from a Pilot Study

Julia Spiekermann^1,2^, Jakob Höppner^1,2^, Eliena Ibnukshein^1^, Kathrin Sinningen^1^, Beatrice Hanusch^1^, Cordula Kiewert^3^, Heide Siggelkow^4^, Corinna Grasemann^1,2^

Corresponding author: Corinna Grasemann, Division of Rare Diseases, University Hospital of Pediatrics and Adolescent Medicine, St. Josef-Hospital, Ruhr-University Bochum, Alexandrinenstraße 5, 44791 Bochum, Germany; Tel.: 0049 (0)234 5096675; E-Mail: [corinna.grasemann@rub.de](mailto:corinna.grasemann@rub.de)

| **Suppl. Table 1: Detailed information of 20 participants** | | | | | | | | | | | | | |
| --- | --- | --- | --- | --- | --- | --- | --- | --- | --- | --- | --- | --- | --- |
| **Study ID** | **Age at visit (years)** | **Age at diagnosis (years)** | **Height (cm)** | **Armspan (cm)** | **BMI (kg/m^2^)** | **Birth height (cm)** | **Birth weight (g)** | **Mother’s height (cm)** | **Father’s height (cm)** | **Testicular volume (ml)** | | **Testosterone Replacement Therapy** | **Current Medication** |
|  |  |  |  |  |  |  |  |  |  | **right** | **left** |  |  |
| 1 | 14,29 | 0 | 172.4 | 171 | 24.6 | n/A | n/A | 172 | 185 | 5 | 5 | / | / |
| 2 | 25,43 | 17 | 178 | n/A | 38.7 | 48 | 3200 | 167 | 178 | 1 | 1 | 250mg i.m./3 weekly | / |
| 3 | 13,40 | 0 | 166.2 | 165.5 | 18.2 | 53 | 3390 | 170 | 183 | 2 | 2 | / | / |
| 4 | 16,37 | 0 | 188.4 | 187 | 17.9 | 52 | 3400 | 165 | 186 | 1 | 1 | 250mg i.m. /4 weekly | / |
| 5 | 11,41 | 7 | 174.7 | 177 | 21.2 | 53 | 3980 | 175 | 187 | 0.2 | 0 | / | / |
| 6 | 24,22 | n/A | 188.4 | 190 | 18.8 | 49 | 3200 | 172 | 190 | n/A | n/A | unknown | celecoxib, pantoprazol |
| 7 | 9,26 | 9 | 138.6 | 138 | 21.1 | 53 | 4250 | 173 | 180 | 0.5 | 0.5 | / | budesonid, omeprazol |
| 8 | 19,84 | 17 | 187 | 189 | 19.2 | 56 | 3840 | 168 | 176 | n/A | n/A | 50mg/daily;  transdermal | / |
| 9 | 15,19 | 0 | 181 | n/A | 19.4 | 51 | 3030 | 164 | 176 | 5 | 5 | / | / |
| 10 | 13,88 | 12 | 177.4 | n/A | 18.2 | 54 | 2920 | 164 | 180 | n/A | n/A | / | methylphenidate acetazolamid, vitamin D |
| 11 | 16,44 | 13 | 192.4 | 194 | 22.7 | 50 | 3645 | 172 | 184 | 3 | 3 | 50 mg/daily, transdermal | / |
| 12 | 20,91 | 4 | 191.3 | 193 | 23.9 | 54 | 4280 | n/A | n/A | 1 | 1 | 50 mg/daily, transdermal | lisdexamfetamine, vitamin D |
| 13 | 19,93 | 11 | 191 | 197 | 18 | 50 | 2840 | 173 | 182 | 5 | 5 | 250mg i.m. /4 weekly | pregabalin |
| 14 | 12,45 | 1 | 158.2 | 159 | 26.1 | 46 | 2490 | 158 | 174 | 6 | 0 | / | macrogol |
| 15 | 14,22 | 0 | 180.8 | 185 | 18.4 | n/A | n/A | 172 | 180 | 2 | 2 | / | risperidone, dexamphetamine |
| 16 | 13,01 | 0 | 176.8 | 180 | 24 | 53 | 3350 | 162 | 188 | 10 | 10 | / | / |
| 17 | 16,61 | n/A | 183.8 | 194 | 23.9 | n/A | n/A | 169 | 170 | 3 | 3 | / | / |
| 18 | 16,39 | n/A | 172.4 | 175 | 26.8 | n/A | n/A | 157 | 173 | 10 | 10 | / | salmeterol/fluticasone, salbutamol, vitamin D |
| 19 | 17,19 | 13 | 179.5 | 185 | 31.5 | 48 | 2750 | 158 | 174 | 2 | 2 | / | / |
| 20 | 10,64 | n/A | 156,5 | 151 | n/A | 57 | 2950 | 162 | 175 | 0,5 | 0,5 | / | lisdexamfetamine |
| ID – identification number; BMI – body mass index | | | | | | | | | | | | | |

| **Suppl. Table 2: Detailed laboratory information of 20 participants** | | | | | | | | | | | | | | | | | | | |
| --- | --- | --- | --- | --- | --- | --- | --- | --- | --- | --- | --- | --- | --- | --- | --- | --- | --- | --- | --- |
| **Study ID** | **Hemo-globin (g/dl)** | **LH (IU/l)** | **FSH (IU/l)** | **Testo-sterone (ng/dl)** | **Cor-tisol (ug/dl)** | **Leptin (ng/ml)** | **25-OHVD (ng/ml)** | **1,25-OHVD (pg/ml)** | **Serum phos-phate (mg/dl)** | **Serum calcium (mmol/l)** | **TSAP (U/l)** | **BAP (ug/l)** | **IGF-1 (ng/ml)** | | **PTH (pg/ml)** | **OC (ng/ml)** | **CTX (pg/ml)** | **Urinary calcium to creatinine ratio (mg/mg)** | **Urinary DPD**  **(ug/g crea-tinine)** |
| 1 | 12,6 | 4,4 | 14,7 | 161 | 15 | 38 | 13,5 | 63,6 | 4,96 | 2,5 | 363 | 119 | 383 | 42,3 | | 49,9 | 1583 |  |  |
| 2 | 16 | 13,4 | 22,4 | 680 | 6,24 | 22 | 14,8 | 57,5 | 3,29 | 2,5 | 84 | 20,6 |  | 21,3 | | 16,3 | 358 |  |  |
| 3 | 13,5 | 4,2 | 11,3 | 168 | 5,23 | 3,7 | 28,4 | 93 | 3,93 | 2,5 | 536 | >240 | 353 | 28 | | 52,5 | 1431 | 0,09 |  |
| 4 | 13,5 | 13,4 | 29,1 | 170 | 6,43 | 1,9 |  | 69,4 | 3,86 | 2,5 | 281 | 111 |  | 20,9 | | 48,1 | 1166 |  | 102 |
| 5 | 12,1 | <0,3 | 1 | <2,50 | 3,07 | 3 | 24,9 | 83,4 | 4,06 | 2,5 | 310 | 117 | 172 | 45 | | 64 | 2013 | 0,20 | 193 |
| 6 | 15,3 | 4,5 | 8,6 | 598 | 5,52 | <0,50 | 30 | 40 | 3,15 | 2,4 | 55 | 11,2 |  | 28,3 | | 18,6 | 642 |  |  |
| 7 | 13,9 | <0,3 | 0,6 | <2,50 | 2,31 | 17 | 24,5 | 58,1 | 4,21 | 2,6 | 260 | 102 | 164 | 39,5 | | 25,7 | 1446 | 0,02 |  |
| 8 | 15,2 | 13,5 | 44,4 | 945 | 4,56 | 2,4 | 26 | 69,3 | 3,87 | 2,5 | 106 | 37,3 |  | 27,6 | | 27,3 | 626 | 0,14 | 43,1 |
| 9 | 14,7 | 17 | 27,7 | 326 | 5,78 | 5,4 | 17 | 84,8 | 4,84 | 2,6 | 293 | 101 |  | 49,4 | | 121 | 2702 |  | 161 |
| 10 | 15,7 | 2,9 | 7,5 | 289 | 3,13 | 5,9 |  | 61,4 | 4,13 | 2,5 | 218 | 81 | 294 | 38,4 | | 50,6 | 2226 | 0,08 |  |
| 11 | 14,7 | 8,5 | 35,6 | 372 | 6,22 | 12 |  | 55,5 | 2,88 | 2,5 | 131 | 36,5 |  | 39,9 | | 35,5 | 985 | 0,08 | 61,1 |
| 12 | 14,2 | 25,7 | 33,7 | 749 | 4,36 | 3,6 | 19 | 40,9 | 4,09 | 2,5 | 71 | 17 |  | 40,7 | | 23,9 | 440 | 0,12 | 28,5 |
| 13 | 14 | <0,3 | 1,3 | 362 | 5,23 | 1,1 | 44,4 | 43 | 3,84 | 2,5 | 101 | 39,6 |  | 19,4 | | 25,4 | 565 | 0,12 | 27,4 |
| 14 | 13,5 | 0,6 | 5,2 | 5,95 | 7,67 | 28 | 25,9 | 85,2 | 4,75 | 2,4 | 266 | 105 |  |  | | 56,1 |  | 0,14 | 242 |
| 15 | 14,8 | 33,1 | 64,1 | 218 | 6,96 | 1,8 | 26,8 | 55,8 | 3,4 | 2,5 | 132 | 44,3 | 445 | 31,7 | | 59,9 | 1352 | 0,09 | 82,4 |
| 16 | 13,2 | 2,2 | 2,4 | 64,9 | 3,59 | 11 | 4,7 | 72,5 | 4,42 | 2,5 | 252 | 106 |  | 65,3 | | 61,4 | 1236 | 0,08 | 183 |
| 17 | 13,9 | 32,2 | 34,1 | 446 | 10,4 | 8 | 25,6 | 66,8 | 3,89 | 2,5 | 121 | 40,3 | 313,1 | 31,2 | | 46,1 | 1127 | 0,01 | 70 |
| 18 | 12,4 | 5,1 | 1,7 | 201 | 3,22 | 31 | 19,2 | 69,3 | 3,87 | 2,5 | 128 | 47,1 | 299,9 | 37,5 | | 31,7 | 874 |  |  |
| 19 | 15,6 | 18 | 34,3 | 351 | 5,69 | 27 | 7,8 | 51,9 | 3,27 | 2,5 | 84 | 25,4 | 332 |  | | 25,9 |  | 0,16 | 38,4 |
| 20 | 14,6 | 0,3 | 1,1 | 250 |  |  | 15,3 |  | 3,808 | 2,56 | 305 |  |  |  | |  |  |  |  |
| ID – identification number; 25-OHVD - 25-OH vitamin D; 1,25-OH2VD – 1,25-OH vitamin D; total serum alkaline phosphatase – TSAP; bone specific alkaline phosphatase – BAP; parathyroid hormone -PTH (pg/ml); osteocalcin – OC; insulin-like growth factor-1 - IGF-; beta-crosslaps - CTX; follicle stimulating hormone – FSH; luteinizing hormone – LH. | | | | | | | | | | | | | | | | | | | |

| **Suppl. Table 3: Inter- and intra-assay precision** | | |
| --- | --- | --- |
| **Laboratory Parameter** | **Intra-assay precision** | **Inter-assay precision** |
| LH | 0.8 % | 1.1 % |
| FSH | 0.4 % | 1.0 % |
| Free testosterone | 1.3 % | 1.4 % |
| Total testosterone | 1.3 % | 1.4 % |
| IGF-1 | 0.8 % | 1.4 % |
| PTH | 0.9 % | 1.0 % |
| Osteocalcin | 1.4 % | 2.0 % |
| 1,25-OHVD | 2.4 % | 7.1 % |
| 25-OHVD | 2.6 % | 3.5 % |
| TSAP | 0.4 % | 1.1% |
| BAP | 4.6 % | 6.4 % |
| CTX | 1.2 % | 1.5 % |
| Leptin | 5.2 % | 12 % |
| Urinary DPD | 1.6 % | 3.5 % |
| LH - Luteinizing hormone; FSH - Follicle-stimulating hormone; IGF-1 - Insulin-like growth factor 1; PTH – Parathyroid hormone; (1,)25-OHVD – (1,)25-OH vitamin D; TSAP – total serum alkaline phosphatase; BAP – bone specific alkaline phosphatase; CTX – beta-crosslaps; urinary DPD – urinary deoxypyridinoline. | | |

Supp. Figure 1: Bone turnover markers in the cohort: a. osteocalcin (ng/ml) in n = 19, b. ß-crosslaps (pg/ml) n = 17 and c. bone specific alkaline phosphatase (BAP) (ug/l) in n = 19 are mostly within age-appropriate norms; grey area indicates the age-appropriate reference ranges (a-c).

**
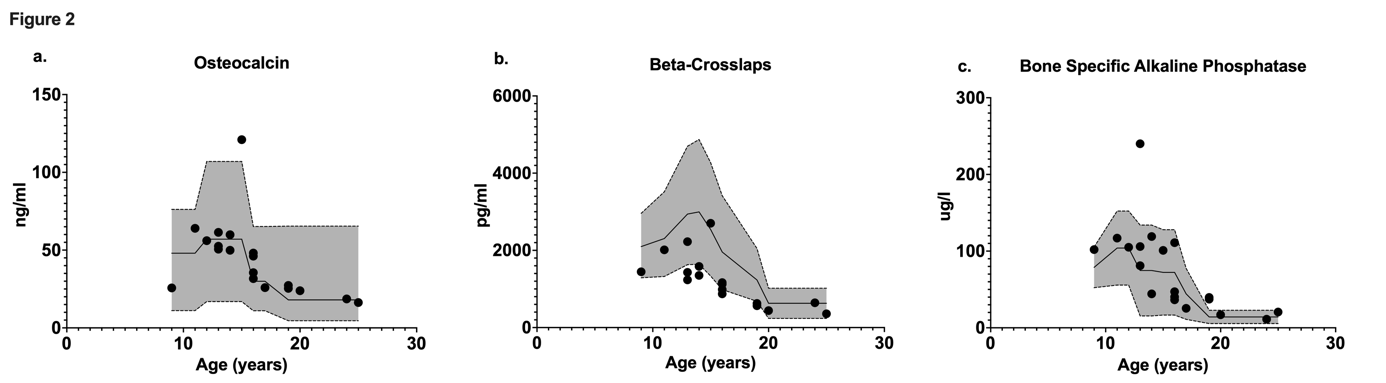
**
